# General vs spinal anesthesia in transvaginal hysterectomy with anterior and posterior repair with pelvic organ prolapse patients: Retrospective cohort study

**DOI:** 10.1097/MD.0000000000048144

**Published:** 2026-04-03

**Authors:** Tae Young Kim, Chul Hong Kim, Moon Kyoung Cho

**Affiliations:** a Department of Obstetrics and Gynecology, Chonnam National University Medical School, Gwangju, Republic of Korea.

**Keywords:** general anesthesia, pelvic organ prolapse, postoperative urinary retention, spinal anesthesia, transvaginal hysterectomy

## Abstract

Pelvic organ prolapse (POP) is commonly managed with transvaginal hysterectomy (TVH) and pelvic floor repair. As the population ages, the need for POP surgery continues to increase, typically under either spinal or general anesthesia. Although spinal anesthesia is associated with fewer postoperative complications, concerns regarding postoperative urinary retention (POUR) remain. This study was designed to investigate whether the type of anesthesia influences the incidence of POUR in POP surgery. This retrospective cohort study analyzed 350 patients who underwent TVH with anterior and posterior repair for POP at the Chonnam National University Hospital between January 2018 and June 2023. Patients were divided into spinal anesthesia (n = 167) and general anesthesia (n = 183) groups. Primary outcomes were POUR, catheter reinsertion, length of hospital stay, and urinary symptoms. Multivariable logistic regression was used to identify independent predictors of post-discharge urinary tract infection symptoms. A total of 350 patients were analyzed (spinal anesthesia, n = 167; general anesthesia, n = 183). The baseline characteristics, including age, body mass index, parity, and comorbidities, were comparable between the groups. No significant differences were observed in POP quantification stage or perioperative variables. Postoperatively, catheter reinsertion was required in 9.0% of the spinal group and 2.2% of the general group (*P* = .182). Urinary symptoms at 1 week occurred in 1.8% and 7.2% of the spinal anesthesia and general anesthesia group, respectively (*P* = .176). Multivariate logistic regression identified no independent predictors of post-discharge urinary tract infection symptoms. Both anesthesia types demonstrated comparable safety, voiding function, and recovery outcomes after TVH with anterior and posterior repair for POP. POUR rates were comparable between the spinal and general anesthesia groups, supporting their similar safety and recovery profiles, consistent with the principles of enhanced recovery after surgery.

## 1. Introduction

Transvaginal hysterectomy (TVH), often combined with pelvic floor repair, remains the standard surgical treatment for pelvic organ prolapse (POP).^[1,2]^ As the population ages and the prevalence of POP increases among older women, the demand for surgical intervention is projected to increase substantially.^[1,3]^ TVH with anterior and posterior repair is typically performed under general anesthesia or regional techniques such as spinal or epidural anesthesia. As the number of older women increases, the incidence of POP and corresponding demand for surgical correction are expected to increase significantly. Perioperative management strategies that minimize postoperative complications have become increasingly important.

POUR is defined as the inability to void after surgery, according to the specific clinical criteria established by the surgeon.^[4]^ This condition affects between 2% and 40% of patients following surgery for POP or stress urinary incontinence, primarily resulting from disruption of the micturition reflex.^[5,6]^ POUR rates are particularly elevated after vaginal surgery including anterior repair, incontinence procedures, and isolated posterior repair.^[7–9]^ Contributing factors include alterations in the ureteral junction angle, denervation, muscular dissection-related changes, and mild urethral obstruction following surgical intervention.^[7,10]^ Complications frequently arise from POUR and include pain, prolonged hospitalization, bladder overdistension, urinary tract infection (UTI) or bacteremia, and autonomic responses that worsen postoperative outcomes.^[11]^ Therefore, strategies to minimize postoperative POUR should be further investigated.

Risk factors for POUR include older age, longer duration of surgery, type of anesthesia used, and baseline bladder issues. The risk is also higher in patients who have undergone prior incontinence surgery or advanced POP. Other contributing factors were postoperative opioid use and UTIs. Specifically, greater opioid use increased the risk of POUR by 1.5 times (odds ratio [OR] = 1.3).^[12,13]^ Spinal anesthesia, while associated with reduced risks of thromboembolic and pulmonary complications, may impair afferent bladder signaling to the pontine micturition center, thereby increasing the likelihood of urinary retention.^[14–18]^ Although multiple studies have investigated the relationship between anesthesia type and POUR, no consensus regarding the optimal postoperative day (POD) for conducting voiding trials. The incidence of urinary retention appears to vary according to surgical procedure type and anesthesia duration.^[6,19]^ Importantly, data specifically evaluating anesthesia-related outcomes in patients undergoing TVH with combined anterior and posterior repair are limited.

Therefore, the primary objective of this study was to compare the incidence of POUR in patients undergoing TVH with anterior and posterior repair under general versus spinal anesthesia. This comparison aims to support informed, evidence-based decision-making and enhance preoperative patient counseling. The secondary objectives were to compare the demographic characteristics of the patients based on the type of anesthesia administered and to describe the utilization patterns of each anesthesia type within the cohort. By achieving these objectives, this study aimed to elucidate how anesthesia choice influences postoperative outcomes and to inform the best practices in the surgical management of POP.

## 2. Methods

### 2.1. Study design and patient selection

**Figure 1.**
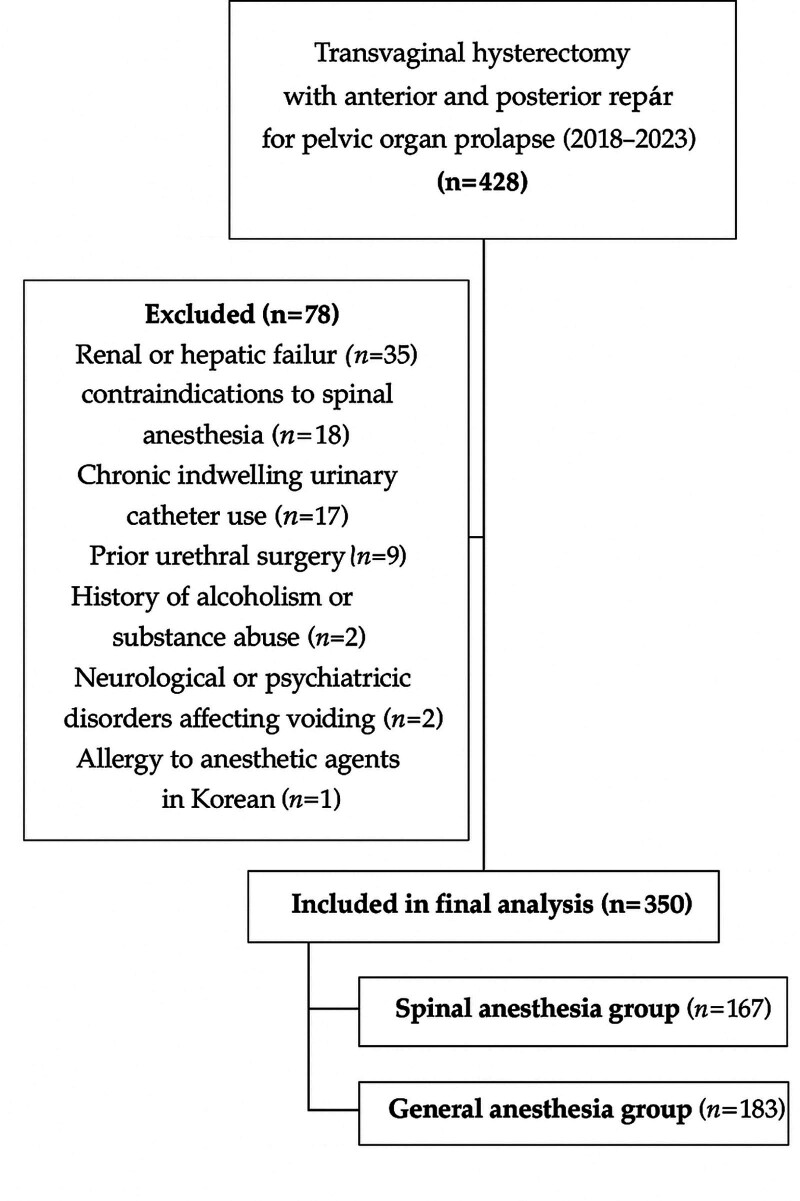
Flow diagram of patient selection. Among 428 women who underwent transvaginal hysterectomy (TVH) with anterior and posterior repair for pelvic organ prolapse (POP) between January 2018 and June 2023, 78 were excluded based on predefined criteria. The remaining 350 patients were included in the final analysis and categorized into the spinal anesthesia group (n = 167) and the general anesthesia group (n = 183). n = number of patients, POP = pelvic organ prolapse, TVH = transvaginal hysterectomy.

This retrospective cohort study was conducted at the Department of Obstetrics and Gynecology of the Chonnam National University Medical Hospital (Gwangju, South Korea). The inclusion criteria were women who underwent TVH combined with anterior and posterior repair for POP between January 2018 and June 2023. A total of 428 patients met the inclusion criteria during the study period. Data were collected from the hospitals’ electronic medical record system. After applying the exclusion criteria, 350 patients were included in the final analysis and stratified by anesthesia type into spinal anesthesia (n = 167) and general anesthesia (n = 183) groups. All consecutive patients who met the inclusion criteria during this study period were included to minimize selection bias. The patient selection process is summarized in Figure 1. The exclusion criteria were renal or hepatic failure, contraindications to spinal anesthesia, chronic indwelling urinary catheter use, prior urethral surgery, history of alcoholism or substance abuse, neurological or psychiatric disorders affecting voiding, allergy to anesthetic agents (e.g., lidocaine, opioids, and bupivacaine), and inability to communicate fluently in Korean.

### 2.2. Ethics statement

This study was conducted in accordance with the principles of the Declaration of Helsinki. The Institutional Review Board approved the study protocol (approval no. CNUH-2025-277), and all patient data were anonymized before the analysis to ensure confidentiality. All investigations were conducted in accordance with the guidelines and regulations of the Institutional Review Board. Owing to its retrospective design, the requirement for written informed consent was waived for all study participants as the research posed minimal risk.

### 2.3. Data collection

Clinical and demographic data included age, body mass index, parity, number of vaginal and cesarean deliveries, American Society of Anesthesiologists physical status classification, history of diabetes mellitus (DM), recurrent UTIs, prior pelvic surgery, smoking status, chronic obstructive pulmonary disease, and chronic steroid use. The POP quantification system was used to assess POP stages.

### 2.4. Surgical technique and anesthesia procedures

All surgeries were performed by a single staff surgeon to minimize interoperator variability. General anesthesia was induced with 100 mg of 1% propofol (Fresofol MCT, Fresenius Kabi, Bad Homburg, Germany) administered intravenously, followed by 50 mg of rocuronium bromide (Esmeron, Merck Sharp & Dohme, Haarlem, Netherlands) to achieve neuromuscular blockade. After confirming the patient’s level of consciousness by using a bispectral index monitor, tracheal intubation was performed. General anesthesia was maintained using total intravenous anesthesia with a continuous intravenous infusion of remifentanil (Remiva, Hana Pharm Co., Ltd., Seoul, Republic of Korea) in combination with propofol throughout the surgical procedure. For spinal anesthesia, the patient was positioned in the lateral decubitus position. After numbing the injection site with 2 mL of 2% lidocaine (Lidocaine HCl, Huons Co., Ltd., Seongnam, Republic of Korea), a small pencil-point needle was inserted into the lower back at the L3/L4 or L4/L5 level via either a midline or paramedian approach, angling the needle bevel toward the lowest point of the spine. A 9 mg dose of hyperbaric 0.5% bupivacaine (Marcaine heavy, Aspen Pharma, Dublin, Ireland) was injected slowly into the subarachnoid space over 1 minute. Sensory blockade was assessed by loss of cold sensation, and motor blockade was evaluated using the Bromage scale. Intravenous midazolam (Midazolam Bukwang, Bukwang Pharmaceutical Co., Ltd., Seoul, Republic of Korea) 3 mg was administered concurrently for sedation. During surgery, patients in both groups were administered 1 L/h of an intravenous plasma solution (Plasma solution A, SK Plasma, Seongnam, Republic of Korea). The urinary drainage bag was emptied before transfer to the recovery room.

### 2.5. Preoperative & Postoperative management

All the patients received preoperative antibiotic prophylaxis with intravenous cefazolin sodium (Cefazolin, Chong Kun Dang Pharmaceutical Corp., Seoul, Republic of Korea). Postoperatively, a urinary catheter was maintained for 3 days and removed on POD 4. Patients were then required to void within 4 hours of catheter removal, after which post-void residual (PVR) urine volume was measured using a bedside bladder scanner (Bicon-700, Mcube Technology Co., Ltd., Seoul, Republic of Korea). POUR was defined as the inability to void within 4 hours of catheter removal or a PVR urine volume of ≥ 200 mL after spontaneous voiding, as measured by bladder ultrasonography. If either of these occurred, the patient was re-catheterized and the catheter was removed again on POD 6. Cystitis was defined based on urinalysis, which revealed either significant bacteriuria ( ≥ 2+) or a positive nitrate result. The treatment regimen for the diagnosed patients consisted of 500 mg oral ciprofloxacin (Cycin tab, Hanmi Pharm Co., Ltd., Seoul, Republic of Korea) twice daily for 5 days. Postoperatively, a patient-controlled analgesia device was provided for rescue pain management. The pump was programmed to deliver 1 mg of intravenous fentanyl citrate (Fentanyl citrate HANA, Hana Pharm Co., Ltd., Seoul, Republic of Korea), nefopam hydrochloride (Allpain inj, BCworld Pharm Co., Ltd., Seoul, Republic of Korea), and ramosetron hydrochloride (Nasea, Astellas Pharma Inc., Tokyo, Japan) on demand, with a 7-minute lockout interval and a 2-hour dose limit of 16 mg. Upon discharge, oral mefenamic acid (Somalgen TAB, Bukwang Pharmaceutical Co., Ltd., Seoul, Republic of Korea) was prescribed for continued analgesia.

### 2.6. Statistical analysis

No formal a priori sample size calculation was performed due to the retrospective nature of the study. Instead, all consecutive eligible patients during the study period were included to maximize statistical power and provide a comprehensive evaluation of available clinical data. Statistical analyses were performed using Statistical Package for the Social Sciences software (version 23.0; IBM Corp., Armonk). The normality of continuous variables was assessed using the Shapiro–Wilk test. Normally distributed variables were analyzed using Student *t* test, whereas non-normally distributed variables were analyzed using Mann–Whitney U test. Categorical variables were compared using Fisher exact test for 2 × 2 comparisons and Pearson χ^2^ test for comparisons involving more than 2 categories. Multivariable logistic regression analysis was subsequently conducted to identify independent predictors of postoperative urinary retention (POUR). Variables with a *P* value < .10 in the univariate analysis were entered into the multivariable model to adjust for potential confounders. Patients with missing data were excluded from analysis. ORs and 95% confidence intervals were calculated, and a 2-tailed *P* < .05 was considered significant.

## 3. Results

### 3.1. Baseline patient characteristics

In total, 350 patients met the inclusion criteria and were included in the final analysis. Patients were divided into 2 groups based on the type of anesthesia they received: the spinal anesthesia group (n = 167) and the general anesthesia group (n = 183). Descriptive statistics were used to present baseline characteristics of the study population. Group differences were evaluated using statistical tests.

The 2 groups were well matched in terms of age, with the mean age of 71.4 ± 8.23 years in the spinal anesthesia group and 70.5 ± 8.96 years in the general anesthesia group (*P* > .547). These differences were not statistically significant, indicating a well-matched obstetric background. There was no significant difference in mean body mass index between the spinal anesthesia group (24.0 ± 2.3) and the general anesthesia group (24.1 ± 2.9) (*P* = .355). Mean parity was also comparable between the 2 groups (3.12 ± 1.2 vs 3.11 ± 1.3; *P* = .945). Regarding obstetric history, a prior cesarean section history was slightly more frequent in the spinal anesthesia group (7.1% [n = 12]) than in the general group (4.9% [n = 8]), although the difference was not statistically significant (*P* = .085). Previous pelvic surgeries, including cesarean sections, myomectomies, salpingo-oophorectomy, and ovarian cystectomies, were reported in 29.5% (n = 49) of the patients in the spinal anesthesia group and 20.4% (n = 37) of the patients in the general anesthesia group (*P* = .246). Analysis of comorbid conditions revealed no statistically significant difference in the prevalence of hypertension (52.5%, n = 87 vs 52.5%, n = 96; *P* = .467) or DM (14.7%, n = 24 vs 23.7%, n = 43; *P* = .212). Other conditions, such as smoking history, chronic obstructive pulmonary disease, and chronic steroid use, were rare and evenly distributed. The American Society of Anesthesiologists physical status classification indicated that the preoperative health profiles of the 2 groups were similar. The groups were comparable across classes I–IV (all *P* > .5) (Table 1).

**Table 1:** Baseline patient characteristics.

Characteristics	Spinal group (n = 167)	General group (n = 183)	*P* value
Age (yr)	71.46 ± 8.23	70.51 ± 8.96	.547
BMI (kg/m^2^)	24.0 ± 2.3	24.1 ± 2.9	.355
Parity	3.12 ± 1.2	3.11 ± 1.3	.945
Number of cesarean sections (%)	7.1 (n = 12)	4.9 (n = 8)	.085
ASA classification			
Class I	49.4% (n = 83)	38.0% (n = 70)	.545
Class II	35.2% (n = 59)	40.7% (n = 74)	.697
Class III	15.4% (n = 25)	20.4% (n = 38)	.589
Class IV	0.0% (n = 0)	0.5% (n = 1)	.552
Previous pelvic surgery	29.5% (n = 49)	20.3% (n = 37)	.246
Medical history			
DM	14.7% (n = 24)	23.7% (n = 43)	.212
Hypertension	52.5% (n = 87)	52.5% (n = 96)	.467
Smoking	10.4% (n = 17)	8.4% (n = 15)	.562
COPD	0.0% (n = 0)	0.3% (n = 1)	.651
Chronic steroid use	0.0% (n = 0)	1.2% (n = 3)	.507

Vaules are presented as Mean ± Standard deviation or (%), as appropriate.

ASA = American Society of Anesthesiologists, BMI *=* body mass index, COPD = chronic obstructive pulmonary disease, DM = diabetes mellitus, kg = kilogram, m = metre, n = number of patients, yr = year.

### 3.2. Primary outcomes: POUR

All patients underwent urinary catheter removal on POD 4. Catheter reinsertion due to POUR was required in 9.0% (n = 15) of patients in the spinal anesthesia group and 2.2% (n = 4) in the general anesthesia group. Although the incidence of catheter reinsertion was higher in the spinal anesthesia group, this difference did not reach statistical significance (*P* = .182). No patients in either group demonstrated evidence of UTI on urinalysis prior to discharge (*P* = .998).

### 3.3. Secondary perioperative and postoperative outcomes

The incidence of preoperative UTI requiring antibiotics treatment was low in both groups (1.8%, n = 3 in the spinal anesthesia group vs 0.0%, n = 0 in the general anesthesia group; *P* = .320). There was no significant difference in the mean length of hospital stay between the spinal anesthesia and general anesthesia groups (4.27 ± 1.4 vs 4.38 ± 1.3; *P* = .564). At the routine outpatient follow-up 1-week after discharge, urinary symptoms such as dysuria, urinary frequency, and urgency were reported more frequently in the general anesthesia group (7.2%, n = 13) than in the spinal anesthesia group (1.8%, n = 3); however, this difference was not statistically significant (*P* = .176). The overall rate of antibiotics prescription for symptomatic patients was low and did not differ significantly between groups (1.8% in the general anesthesia group vs 0.0% in the spinal anesthesia group) (Table 2).

**Table 2:** Perioperative and follow-up outcomes.

Variable	Spinal group (n = 167)	General group (n = 183)	*P* value
Preoperative UTI	1.8% (n = 3)	0.0% (n = 0)	.320
Urinary catheter reinsertion	9.0% (n = 15)	2.2% (n = 4)	.182
POD 4 UTI	0.0% (n = 0)	0.0% (n = 0)	.998
Hospital stay (d)	4.27 ± 1.4	4.38 ± 1.3	.564
OP 1-wk urinary symptoms	1.8% (n = 3)	7.2% (n = 13)	.176
OP 1-wk UTI	0.0% (n = 0)	1.8% (n = 3)	.320

d = day, n = number of patients, OP = operation, POD = postoperative day, UTI = urinary tract infection, wk = week.

### 3.4. POP stage

POP severity was assessed using the POP quantification system. Baseline prolapse severity was comparable between the spinal anesthesia and general anesthesia groups. No statistically significant differences were observed in the distribution of apical, anterior, or posterior compartment stages between the groups (all *P* > .4) (Table 3).

**Table 3:** Pelvic organ prolapse quantification (POP-Q).

Prolapse variable	Spinal group (n = 167)	General group (n = 183)	*P* value
Apical	41.9% (n = 70)	45.9% (n = 84)	
Stage IIc	5	2	.459
Stage IIIc	23	25	.836
Stage IVc	42	57	.876
Anterior	49.1% (n = 82)	49.7% (n = 91)	
Stage IIBa	7	10	.407
Stage IIIBa	75	81	.610
Posterior	8.9% (n = 15)	4.3% (n = 8)	
Stage IIBp	9	5	
Stage IIIBp	6	3	.509

n = number of patients, POP-Q = pelvic organ prolapse quantification.

### 3.5. Logistic regression analyses

Multivariable logistic regression analysis was performed to identify the factors associated with postoperative urinary outcomes. As shown in Table 4, no clinical, operative, or postoperative variables were independently associated with postoperative UTI symptoms. Spinal anesthesia was associated with a lower, but statistically insignificant, risk of postoperative urinary symptoms compared with general anesthesia (OR = 0.24, 95% CI: 0.03–2.23; *P* = .209). No significant associations were observed for age (OR = 1.00, 95% CI: 0.90–1.11; *P* = .948), parity (OR = 0.92, 95% CI 0.43–1.97; *P* = .837), hypertension (OR = 0.67, 95% CI 0.11–4.16; *P* = .664), or DM (OR = 0.53, 95% CI 0.12–3.58; *P* = .574). Additional perioperative factors, including POP stage ≥ 2 (OR = 0.72, 95% CI 0.08–6.75; *P* = .774), prior pelvic surgery stay (OR = 0.32, 95% CI 0.03–2.92; *P* = .309), and length of hospital stay (OR = 1.07, 95% CI 0.60–1.91; *P* = .820), were also not significantly associated with postoperative urinary outcomes.

**Table 4:** Multivariable logistic regression analysis of risk factors for post-discharge UTI symptoms.

Variable	OR (95% CI)	*P* value
Spinal anesthesia (vs general anesthesia)	0.24 (0.03–2.23)	.209
Age (yr)	1.00 (0.90–1.11)	.948
Parity	0.92 (0.43–1.97)	.837
Hypertension	0.67 (0.11–4.16)	.664
DM	0.53 (0.12–3.58)	.574
Previous pelvic surgery	0.32 (0.03–2.92)	.309
POP stage ≥ 2	0.72 (0.08–6.75)	.774
Hospital stay (d)	1.07 (0.60–1.91)	.820

Variables with *P* < .1 in univariable analysis were included in the multivariable model.

CI = confidence interval, d = days, DM = diabetes mellitus, OR= odds ratio, POP = pelvic organ prolapse, UTI = urinary tract infection, yr = year.

## 4. Discussion

This retrospective cohort study evaluated whether the type of anesthesia influences POUR and recovery outcomes in patients undergoing TVH with anterior and posterior repair for POP. The principal finding of this study is that spinal anesthesia was not associated with an increased incidence of POUR, catheter reinsertion, or postoperative urinary symptoms compared with general anesthesia. These results indicate that, when standardized surgical and postoperative management protocols are applied, both anesthesia techniques demonstrate comparable perioperative safety and functional recovery profiles.

Spinal anesthesia has traditionally been regarded as a potential risk factor for POUR, particularly in pelvic and gynecological surgeries. Previous studies have suggested that neuraxial blockade may impair detrusor muscle activity, delay recovery of bladder sensation, and contribute to voiding difficulties, especially in elderly patients.^[20]^ However, in the present study, this theoretical concern was not supported by clinical outcomes. In our retrospective cohort of 350 patients who underwent TVH with anterior and posterior repair for POP, the incidence of POUR did not differ significantly between patients receiving spinal anesthesia and those receiving general anesthesia, despite the advanced age and comorbidity burden of the study population.

Several factors may explain the lack of association between spinal anesthesia and POUR observed in this study. First, all eligible patients were consecutively included, and uniform surgical and postoperative care protocols were applied, minimizing variability related to perioperative management. Second, patients with known predisposing factors for urinary retention- such as neurologic disorders, prior urethral surgery, or chronic catheter dependence- were excluded, thereby reducing baseline risk. Third, a standardized bladder management protocol, including scheduled catheter removal and objective PVR volume assessment, was consistently implemented across both anesthesia groups. In addition, limited postoperative opioid use may have mitigated opioid-related suppression of parasympathetic bladder activity.

Previous studies investigating the relationship between anesthesia type and POUR in gynecologic surgery have yielded inconsistent results. Some reports have demonstrated no increased risk of POUR with spinal anesthesia, particularly when perioperative care in standardized.^[21]^ Other studies have identified higher retention rates in specific subgroups, such as younger women, patients with advanced cystocele, or those with metabolic comorbidities. Taken together, these findings suggest that the development of POUR is likely influenced by multiple interacting factors rather than anesthesia type alone.

The findings of this study are consistent with the general principles of enhanced recovery after surgery (ERAS), which emphasize multimodal analgesia, early mobilization, and functional recovery. Regional anesthesia techniques, including spinal anesthesia, may support these goals by reducing systemic opioid exposure and facilitating early postoperative recovery.^[22–24]^ However, this study was not designed to directly evaluate ERAS outcomes, and the relevance of ERAS protocols should therefore be interpreted as supportive rather than definitive.

Beyond urinary outcomes, spinal anesthesia has been associated with favorable systemic effects in selected patient populations, including reduced pulmonary complications and improved hemodynamic stability. Nevertheless, the choice of anesthesia for vaginal hysterectomy should remain individualized, taking into account patient comorbidities, anticipated operative duration, patient preference, clinician expertise, and institutional resources.

Preoperative patient education may also contribute to consistent postoperative outcomes. In this study, all patients received standardized counseling regarding anesthetic options, postoperative expectations, and bladder management protocols. This approach may have enhanced adherence to postoperative care pathways and reduced variability in recovery outcomes across anesthesia groups.

This study has several limitations. Its retrospective design introduces the possibility of selection bias and residual confounding, despite the use of strict eligibility criteria and standardized protocols. Certain variables such as: intraoperative fluid administration, duration of anesthesia, and subtle neurologic differences could not be fully assessed. In addition, POUR assessment relied primarily on catheter reinsertion and objective measures, while subjective voiding symptoms were not systematically collected. Prospective randomized studies are needed to confirm these findings and to evaluate long-term functional and patient-reported outcomes.

## 5. Conclusion

This study demonstrated that spinal anesthesia was not associated with an increased risk of POUR in patients undergoing TVH with anterior and posterior repair for POP. These findings address previous concerns regarding urinary complications associated with regional anesthesia in gynecologic surgery. Within the context of standardized perioperative management, spinal anesthesia appears to be a safe and effective alternative to general anesthesia for this patient population. Its potential advantages, including reduced opioid requirements, earlier mobilization, and favorable cardiopulmonary profiles, may support its use in appropriately selected patients. Further prospective studies are warranted to confirm these findings and to evaluate long-term functional outcomes.

## Acknowledgments

We sincerely thank Ms. Cho Hee Hwang for her valuable assistance in data analysis.

## Author contributions

**Conceptualization:** Moon Kyoung Cho.

**Data curation:** Tae Young Kim.

**Formal analysis:** Tae Young Kim.

**Investigation:** Chul Hong Kim.

**Methodology:** Chul Hong Kim.

**Supervision:** Moon Kyoung Cho.

**Writing – original draft:** Tae Young Kim.

**Writing – review & editing:** Chul Hong Kim.
